# Dromedary camels as a natural source of neutralizing nanobodies against SARS-CoV-2

**DOI:** 10.1172/jci.insight.145785

**Published:** 2021-03-08

**Authors:** Lotfi Chouchane, Jean-Charles Grivel, Elmoubasher Abu Baker Abd Farag, Igor Pavlovski, Selma Maacha, Abbirami Sathappan, Hamad Eid Al-Romaihi, Sirin W.J. Abuaqel, Manar Mahmoud Ahmad Ata, Aouatef Ismail Chouchane, Sami Remadi, Najeeb Halabi, Arash Rafii, Mohammed H. Al-Thani, Nico Marr, Murugan Subramanian, Jingxuan Shan

**Affiliations:** 1 Department of Microbiology and Immunology, Weill Cornell Medicine, New York, New York, USA.; 2 Genetic Intelligence Laboratory, Weill Cornell Medicine–Qatar, Qatar Foundation, Doha, Qatar.; 3 Department of Genetic Medicine, Weill Cornell Medicine, New York, New York, USA.; 4 Deep Phenotyping Core, Research Branch, Sidra Medicine, Doha, Qatar.; 5 Department of Communicable Diseases Control, Ministry of Public Health, Doha, Qatar.; 6 Department of Immunology, Research Branch, Sidra Medicine, Doha, Qatar.; 7 Laboratoire CYTOPATH, Sousse, Tunisia.; 8 Ministry of Public Health, Doha, Qatar.

**Keywords:** COVID-19, Antigen, Immunotherapy, Peptides

## Abstract

The development of prophylactic and therapeutic agents for coronavirus disease 2019 (COVID-19) is a current global health priority. Here, we investigated the presence of cross-neutralizing antibodies against severe acute respiratory syndrome coronavirus 2 (SARS-CoV-2) in dromedary camels that were Middle East respiratory syndrome coronavirus (MERS-CoV) seropositive but MERS-CoV free. The tested 229 dromedaries had anti–MERS-CoV camel antibodies with variable cross-reactivity patterns against SARS-CoV-2 proteins, including the S trimer and M, N, and E proteins. Using SARS-CoV-2 competitive immunofluorescence immunoassays and pseudovirus neutralization assays, we found medium-to-high titers of cross-neutralizing antibodies against SARS-CoV-2 in these animals. Through linear B cell epitope mapping using phage immunoprecipitation sequencing and a SARS-CoV-2 peptide/proteome microarray, we identified a large repertoire of *Betacoronavirus* cross-reactive antibody specificities in these dromedaries and demonstrated that the SARS-CoV-2–specific VHH antibody repertoire is qualitatively diverse. This analysis revealed not only several SARS-CoV-2 epitopes that are highly immunogenic in humans, including a neutralizing epitope, but also epitopes exclusively targeted by camel antibodies. The identified SARS-CoV-2 cross-neutralizing camel antibodies are not proposed as a potential treatment for COVID-19. Rather, their presence in nonimmunized camels supports the development of SARS-CoV-2 hyperimmune camels, which could be a prominent source of therapeutic agents for the prevention and treatment of COVID-19.

## Graphical abstract

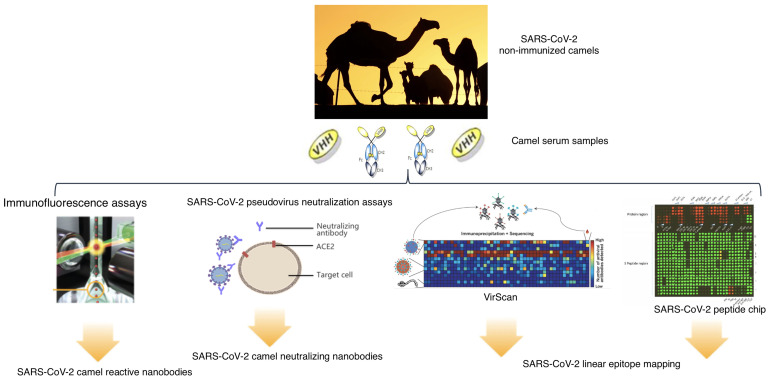

## Introduction

Coronavirus disease 2019 (COVID-19), caused by severe acute respiratory syndrome coronavirus 2 (SARS-CoV-2), is an increasing global threat to public health and economic development. SARS-CoV-2 differs from SARS-CoV and Middle East respiratory syndrome coronavirus (MERS-CoV) by its rapid spread and virulent human-to-human transmission (1). Similar to its 2 predecessors, SARS-CoV-2 is a zoonotic virus, and there is a possibility that it has the same natural reservoir (bats) as SARS-CoV and MERS-CoV (2), with an unknown intermediate host (3).

Although SARS-CoV-2 vaccine development is progressing at a rapid pace, widespread vaccine availability must overcome various hurdles, including antigenic variation, low efficacy, and short-term immune responses (4). Until herd immunity against SARS-CoV-2 develops within communities, preferably by means of effective vaccines, the global population will remain at risk, and health care systems will continue to endure tremendous strain. Novel therapeutic and preventive approaches are being designed and tested worldwide. Passive antibody administration through the transfusion of plasma collected from donors who have recovered from COVID-19, known as COVID-19 convalescent plasma (CCP), has emerged as a promising therapy for the treatment of the disease (5). However, the potential benefits of CCP therapy are hampered by the short-term efficacy of the human polyclonal antibodies, the challenges of scaling up this intervention owing to the unavailability of large amounts of convalescent plasma, the difficulty of mass production, and affordability (6). An attractive alternative would be the use of animal-derived polyclonal antibody therapy, which has been successfully and safely applied in several human conditions (7, 8). An example of this approach is the life-saving postexposure prophylaxis against the rabies virus (9, 10). Animal polyclonal antibody products can be made cost-effectively in large quantities, which makes them suitable for responding to high endemic demand in low-income countries. Importantly, these products may be valuable in addressing a pandemic situation such as the current COVID-19 outbreak.

Several studies have found that dromedary camels are the only intermediate host of MERS-CoV, showing solely asymptomatic juvenile infection (11–15). MERS-CoV has been isolated from the nasal swabs of young dromedaries (11–13), but despite extensive virus screening, MERS-CoV has not been recovered from adult dromedaries (11, 12). MERS-CoV seropositivity rates increase with age to a seroprevalence of nearly 100% in adult dromedaries. In contrast to that in humans, MERS-CoV–exposed dromedary camels develop no disease and show only mild clinical respiratory signs (16–18). The absence of MERS-CoV in adult dromedary camels is correlated with a dramatically potent virus-neutralizing antibody response (16). These findings strongly suggest that dromedary immune system components have the ability to efficiently limit MERS-CoV infection. The strong antibody response observed in dromedaries could be caused by repeated exposure to the virus. However, some studies have attributed this response to a characteristic of dromedaries (17–19), which produce relatively unique heavy homodimeric chain-only antibodies as well as conventional heterotetrameric antibodies. The antigen-binding region of these homodimeric heavy chain–only antibodies consists of 1 single domain, called the VHH. VHHs offer several advantages over common, full-sized antibodies and currently used antibody-based fragments (Fabs, scFvs). These advantages include high specificity, stability, and solubility, as well as a small size, which allows them to recognize unusual antigenic sites and to deeply penetrate tissues. Since their discovery, VHHs have been extensively used in diagnostics and therapies (20–23).

The highly proficient dromedary camel immune system against MERS-CoV and the close structural and functional similarities between the different coronavirus species led us to hypothesize that MERS-CoV–seropositive dromedary camels might have cross-reactive and cross-neutralizing antibodies against SARS-CoV-2. We first assessed the dromedary seroprevalence of antibodies against major SARS-CoV-2 proteins and evaluated their ACE2 binding, inhibitory, and pseudovirus-neutralizing effects. A systematic assessment of the immunodominant B cell antigen determinants of *Betacoronaviruses* among camels and the degree to which these immunodominant B cell targets represent cross-reactive antigenic sites is lacking. Therefore, using high-throughput technologies, we extended our work to include a comprehensive analysis of human and animal virus cross-reactive camel antibody specificities, with an emphasis on the SARS-CoV-2 spike glycoprotein–specific (S-specific) camel cross-reactive VHH antibody repertoire.

## Results

### Identification of SARS-CoV-2 cross-reactive and cross-neutralizing antibodies in dromedaries.

**Figure 1:**
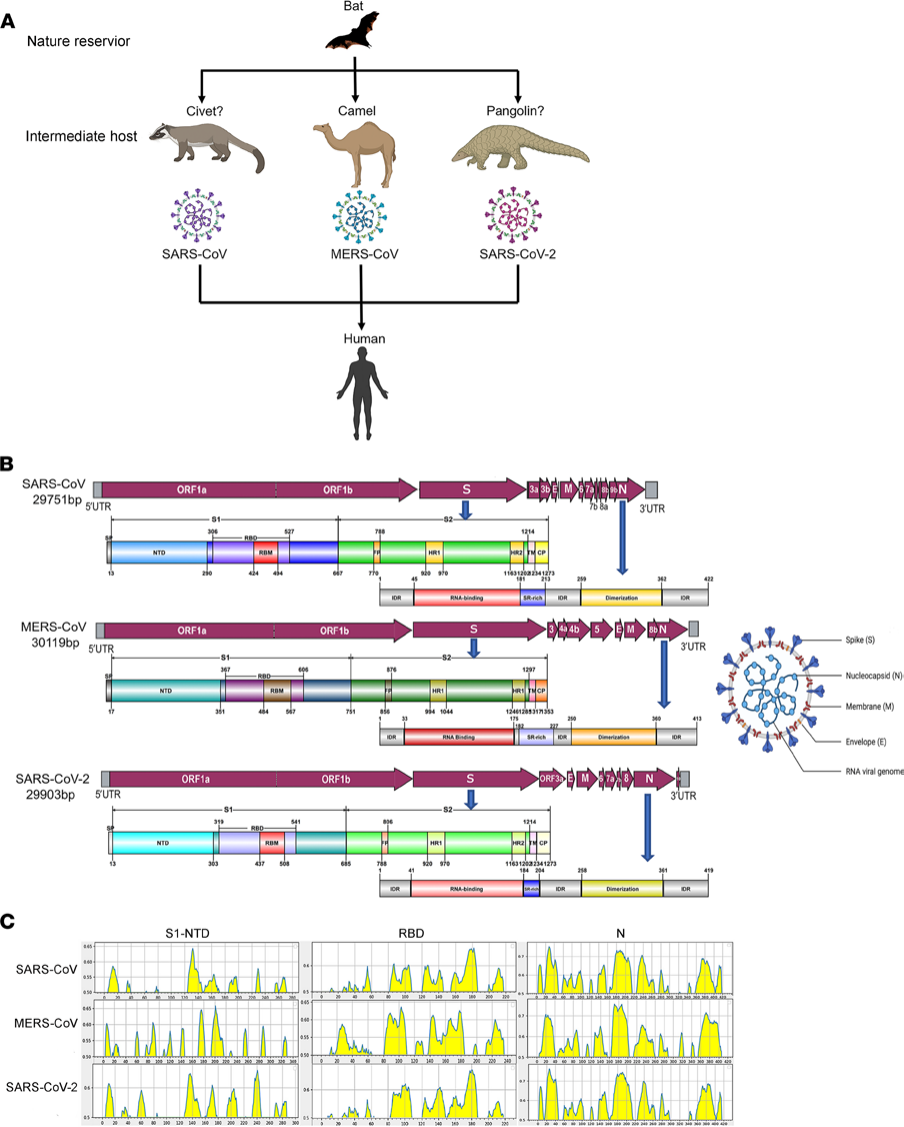
Transmission, structural, and functional homologies of 3 *Betacoronaviruses* (SARS-CoV, MERS-CoV, and SARS-CoV-2). (**A**) Emergence of coronaviruses pathogenic for humans from ancestral bat viruses. (**B**) Schematic representation of the genome organization and functional domains of spike glycoprotein and nucleoprotein proteins for SARS-CoV, MERS-CoV, and SARS-CoV-2. The single-stranded RNA genomes of SARS-CoV, MERS-CoV, and SARS-CoV-2 include 2 large genes, the ORF1a and ORF1b genes, which encode 16 nonstructural proteins (nsp1–nsp16) that are highly conserved throughout coronaviruses. The structural genes encode the structural proteins, spike (S), envelope (E), membrane (M), and nucleocapsid (N), which are common features to all coronaviruses. Other accessory genes are unique to different coronaviruses in terms of number, genomic organization, sequence, and function. The structure of each S and N protein is shown beneath the genome organization. The S protein mainly contains the S1 and S2 subunits. The residue numbers in each region represent their positions in the S or N protein, respectively. CP, cytoplasm domain; IDR, intrinsically disordered region; FP, fusion peptide; HR, heptad repeat; NTD, N-terminal domain; RBD, receptor-binding domain; RBM, receptor-binding motif; SP, signal peptide; SR-rich, serine and arginine rich; TM, transmembrane domain. (**C**) The linear epitope B prediction of spike glycoprotein (S) NTD and RBD and nucleoprotein protein (N) of SARS-CoV, MERS-CoV, and SARS-CoV-2. The peak highlighted in yellow represents the predicted linear epitope by BLEP 2.0 software. The motifs of RBD and N are highly similar among the 3 viruses.

Although the intermediate hosts of SARS-CoV-2, SARS-CoV, and MERS-CoV are different, bats are the key natural reservoir of these 3 viruses, and humans are their final host (Figure 1A). Phylogenetic and structural studies have revealed that SARS-CoV-2, which belongs to the genus *Betacoronavirus*, has a positive sense, approximately 30-kilobase single-standard RNA genome that has considerable similarity to the genomes of SARS-CoV (79%) and MERS-CoV (54%) (Table 1). The striking homology of functional domains and epitope motifs between MERS-CoV and SARS-CoV-2 viral structural proteins highlights the possibility that dromedary MERS-CoV antibodies cross-react with SARS-CoV-2 proteins (Table 1 and Figure 1, B and C). To gain deeper insight into dromedary camel SARS-CoV-2 cross-reactive antibodies, we analyzed serum samples from 229 dromedaries, of which 129 were collected prior to the COVID-19 outbreak (to rule out any hypothetical SARS-CoV-2 interference); the remaining 100 samples were recent and traceable. No cases of dromedary camels carrying SARS-CoV-2 have been reported by the Ministry of Public Health of the State of Qatar, despite extensive screening for the presence of SARS-CoV-2 in domestic animals, including dromedary camels.

**Figure 2:**
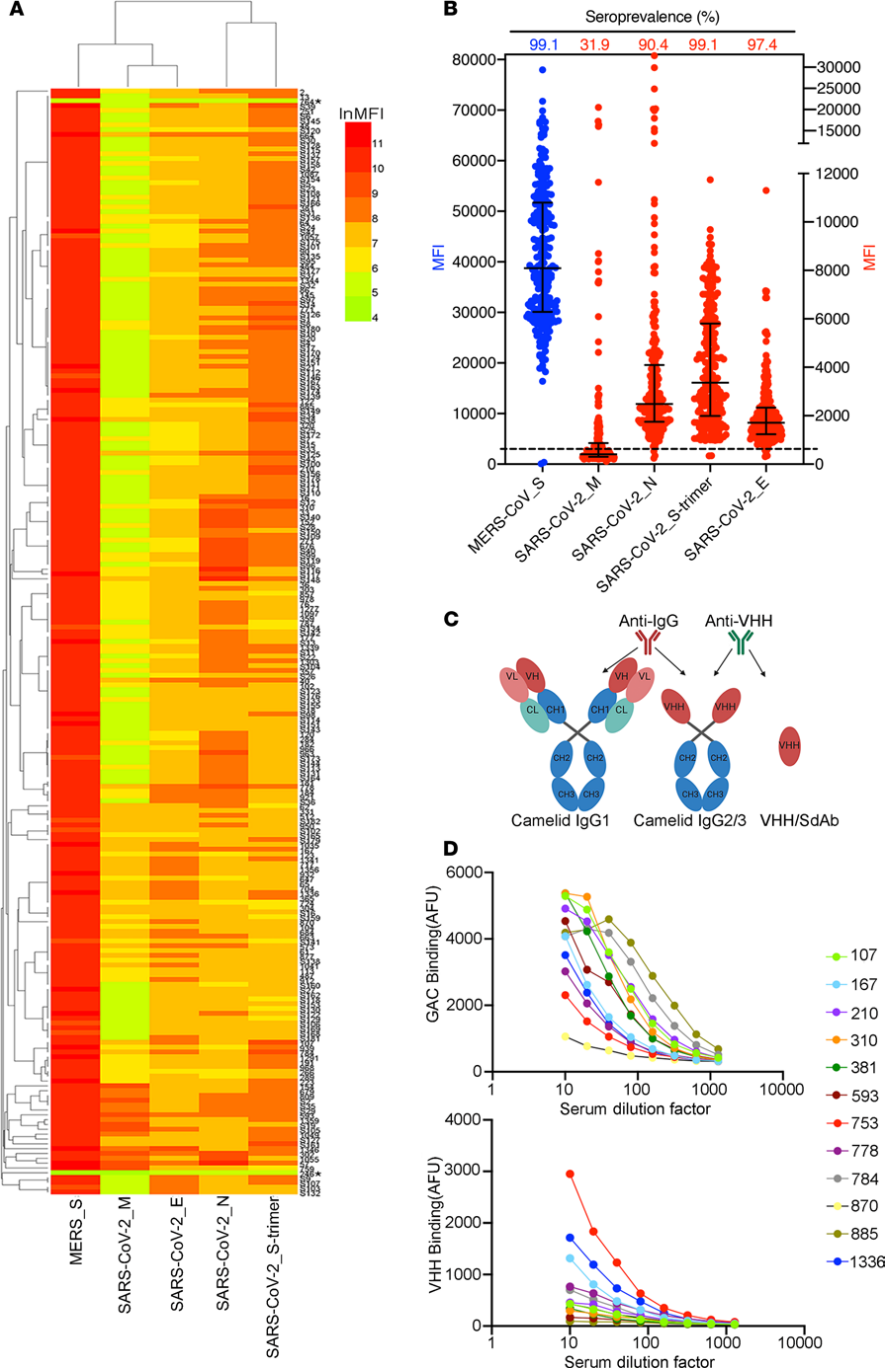
Seroreactivity of dromedary camels to MERS-CoV S protein and SARS-CoV-2 proteins. (**A**) Antibody-binding activities of 229 dromedary sera diluted at 1:20. The asterisks represent serological negative samples that are below 2 times the value of beads only. Each rectangle indicates the camel serum (rows) reactivity to MERS-CoV S protein and to SARS-CoV-2 proteins (columns). Mean fluorescence intensity (MFI) is shown by a color gradient scale. (**B**) The seroprevalence and distribution of antibody-binding activities (mean with interquartile range) of 229 serum samples to each protein. The dashed line indicates the baseline, which is 2 times the value of beads only. (**C**) Schematic structure of IgG1, IgG2/3, and VHH/SdAb. Anti-IgG antibody can recognize total camel IgG antibodies, whereas anti-VHH antibody can only recognize heavy chain alone antibody, IgG2/3, and SdAb. SdAb, single-domain antibody, also known as nanobody. (**D**) SARS-CoV-2 S trimer binding curves for 12 camel sera revealed by anti-IgG camel antibodies (top) and anti-VHH antibodies (bottom), both of which are conjugated with fluorochrome. The sera were diluted 10 times and then subjected to 7-step, 2-fold series dilutions. Each datum point represents the median of up to 500 individual beads. GAC, goat anti-camel IgG; AFU, arbitrary fluorescence units.

We used a highly sensitive fluorescent bead–based immunoassay that takes advantage of the high dynamic range of fluorescent molecule detection by flow cytometry. In this assay, biotinylated MERS-CoV S protein and SARS-CoV-2 proteins were immobilized on polystyrene carboxylated beads to detect cross-reactive antibodies in the dromedary sera. In agreement with previous reports, 227 of the 229 dromedary serum samples (99%) were found to be highly seroreactive to MERS-CoV S protein, with a minimum value approximately 100 times greater than that of beads alone (Figure 2A). The fluorescence values of the 2 negative serum samples were less than 2 times that of beads alone (Figure 2B). Strikingly, all 227 of the positive dromedary serum samples displayed variable but substantial degrees of serological reaction against the SARS-CoV-2 S trimer protein (Figure 2B). Over 90% of the serum samples contained antibodies that bound to the SARS-CoV-2 N and E proteins, and approximately 32% of the samples showed binding to the SARS-CoV-2 M protein (Figure 2B). Importantly, 15% to 30% of the animals exhibited relatively high binding activities to the S trimer and N proteins (Figure 2B). High titers of SARS-CoV-2 S trimer-cross-reactive antibodies were detected in several of the serum samples (Figure 2B). To investigate which antibody subclass the cross-reactivity originated from, anti-camel antibodies that recognize total IgG antibodies or that recognize only VHH antibodies were used to reveal the binding of the camel serum to SARS-CoV-2 S protein (Figure 2C). Interestingly, in some of the serum samples, the SARS-CoV-2 S trimer cross-reactivity predominantly resulted from VHH antibody binding (Figure 2D).

**Figure 3:**
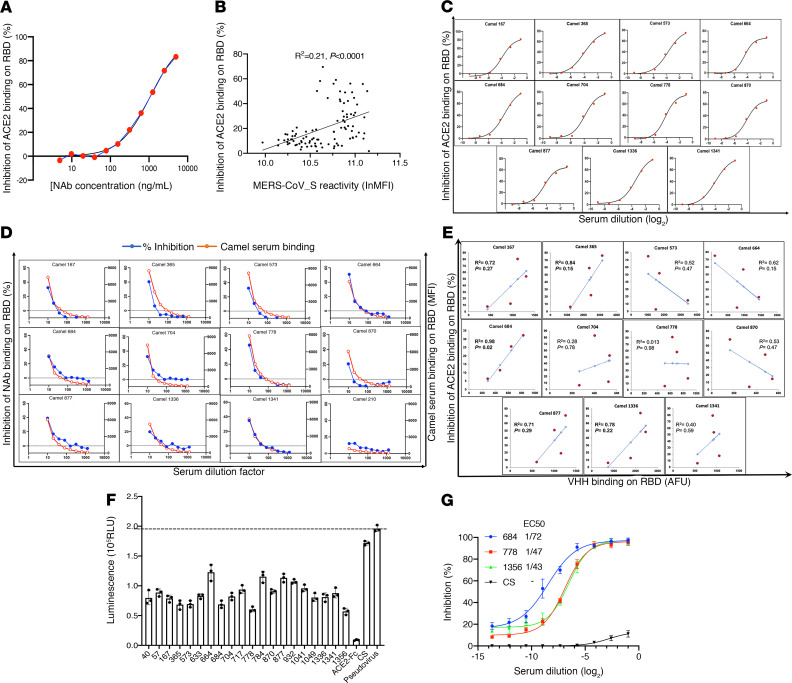
Analysis of virus-neutralizing potential of SARS-CoV-2 cross-reactive camel sera. (**A**) SARS-CoV-2 RBD competition immunofluorescence assay using human neutralizing antibody (NAb) against SARS-CoV-2. The curve started with [NAb] concentration at 5 μg/ml and proceeded in a 12-step, 2-fold dilution series. The [NAb] concentration versus inhibition curve was fit by a 4-parameter logistic model. (**B**) The correlation between RBD binding inhibitory effects and seroreactivity with MERS-CoV S protein in camel sera. MERS-CoV S seroreactive–positive sera from 98 living camels were included. (**C**) Inhibition of ACE2 binding to SARS-CoV-2 RBD by 11 camel sera. The sera were serially diluted to 1:2, 1:6, 1:18, 1:54, 1:162, and 1:486. Each datum point represents the median of up to 500 individual beads. The log_2_(dilution) versus inhibition curves were fit by a 4-parameter logistic model. (**D**) Competition for RBD binding between RBD-specific human IgG1 monoclonal antibody AS35 and camel serum. Camel antibody binding on RBD was revealed by AF594-labeled goat anti-camel antibodies, and human NAb binding was revealed with AF488-labeled goat anti-human anti-IgG1 antibodies. (**E**) The correlation between inhibition of ACE2 binding and VHH antibody binding activity on RBD in 11 camel sera. (**F**) Single-dose (1:50 dilution) pseudovirus neutralizing assay. Twenty camel sera were randomly selected from the ones showing RBD binding inhibitory effects. The virus entry into HEK293 cells was monitored by relative luminescence (RLU). ACE2-Fc, ACE2-conjugated with Fc domain of human IgG; CS, control serum from a healthy individual. Error bars represent the standard deviation of biological triplicates. (**G**) Inhibition curves for 3 sera ranked top in single-dose assay. The sera were diluted 2 times and then subjected to 8-step, 2-fold series dilutions. Typical 4-parameter inhibition curves were observed between log-transformed dilution and inhibition rate (percentage) and were used to determine EC_50_. Error bars represent the standard deviation of biological triplicates.

To further evaluate the presence of SARS-CoV-2 cross-neutralizing antibodies in the camel sera, we established a microsphere-based SARS-CoV-2 competition/inhibition assay to monitor the binding of labeled ACE2 to beads conjugated with S protein or receptor-binding domain (RBD) in the presence or absence of camel sera. The assay was validated by demonstrating an inhibitory effect of an anti–SARS-CoV-2 human neutralizing antibody isolated from a SARS-CoV-2–infected patient. This antibody targeted the RBD of the S protein (Figure 3A), which indicated that this assay could detect antibodies that block SARS-CoV-2 S protein–ACE2 binding in vivo. We used this assay to test the inhibitory effect of 100 camel serum samples. At a 10-fold dilution, nearly 70% of the samples showed obvious inhibitory activity of S protein–ACE2 binding. Interestingly, the camel sera reactivity to MERS-CoV S protein was significantly correlated with their SARS-CoV-2 cross-neutralizing potential (Figure 3B). We also performed a multidose inhibition assay with 11 serum samples, which showed a greater than 50% inhibition efficacy in a single-dose competition/inhibition assay. S-RBD–specific cross-neutralizing antibodies were detectable in up to 50-fold serial dilutions of these 11 serum samples, indicating high specificity and sensitivity (Figure 3C). Next, we examined whether camel serum samples compete with the above-mentioned patient-derived human neutralizing antibody for RBD binding. As shown in Figure 3D, the 11 camel RBD-specific cross-neutralizing antibodies showed variable and partial binding inhibitory effects of the human neutralizing antibody on RBD protein (inhibition ranging between 20% and 50%). This result suggests that these camel sera react with this particular conformational epitope revealed by the human neutralizing antibody. Moreover, this epitope could be also a neutralizing immunodominant epitope, as it is reactive with several neutralizing antibodies. Other camel sera, reacting with SARS-CoV-2 S protein, including serum 210, did not react with this conformational epitope (Figure 3D). To determine whether VHH antibodies play a role in the cross-neutralizing activity, we simultaneously detected VHH antibody–RBD binding and ACE2-RBD binding. VHH–S-RBD binding was highly correlated with the inhibition of ACE2-RBD binding in sera from camels 167, 365, 684, 877, and 1336 (R^2^ > 0.7) (Figure 3E). In camel 684, the inhibitory activity was significantly associated with VHH antibodies (R^2^ = 0.98, *P* = 0.02).

To confirm the results of the camel SARS-CoV-2–neutralizing antibody screening obtained by the microsphere-based SARS-CoV-2 competition/inhibition assay, we applied a cell-based SARS-CoV-2 spike pseudovirus neutralization assay to assess the presence of antibodies preventing pseudovirus entry into host cells in dromedaries showing high titers of SARS-CoV-2 cross-reactive antibodies. A pseudoparticle-based model is a useful tool for evaluating the efficacy of vaccine or antibody candidates against viruses (24–30). All tested camel sera showed medium-to-high titers of SARS-CoV-2 cross-neutralizing antibodies by inhibiting pseudotyped luciferase SARS-CoV-2 spike entry into ACE2-expressing cells, whereas healthy human serum did not (Figure 3F). The top 3 cross-neutralizing serum samples were further analyzed in a multidose assay. The 3 samples showed high neutralizing potency against the SARS-CoV-2 pseudovirus, with an EC_50_ range of 1:40 to 1:70 serum dilution (Figure 3G).

### Epitope mapping of SARS-CoV cross-reactive dromedary camel antibodies using phage immunoprecipitation sequencing (VirScan).

**Figure 4:**
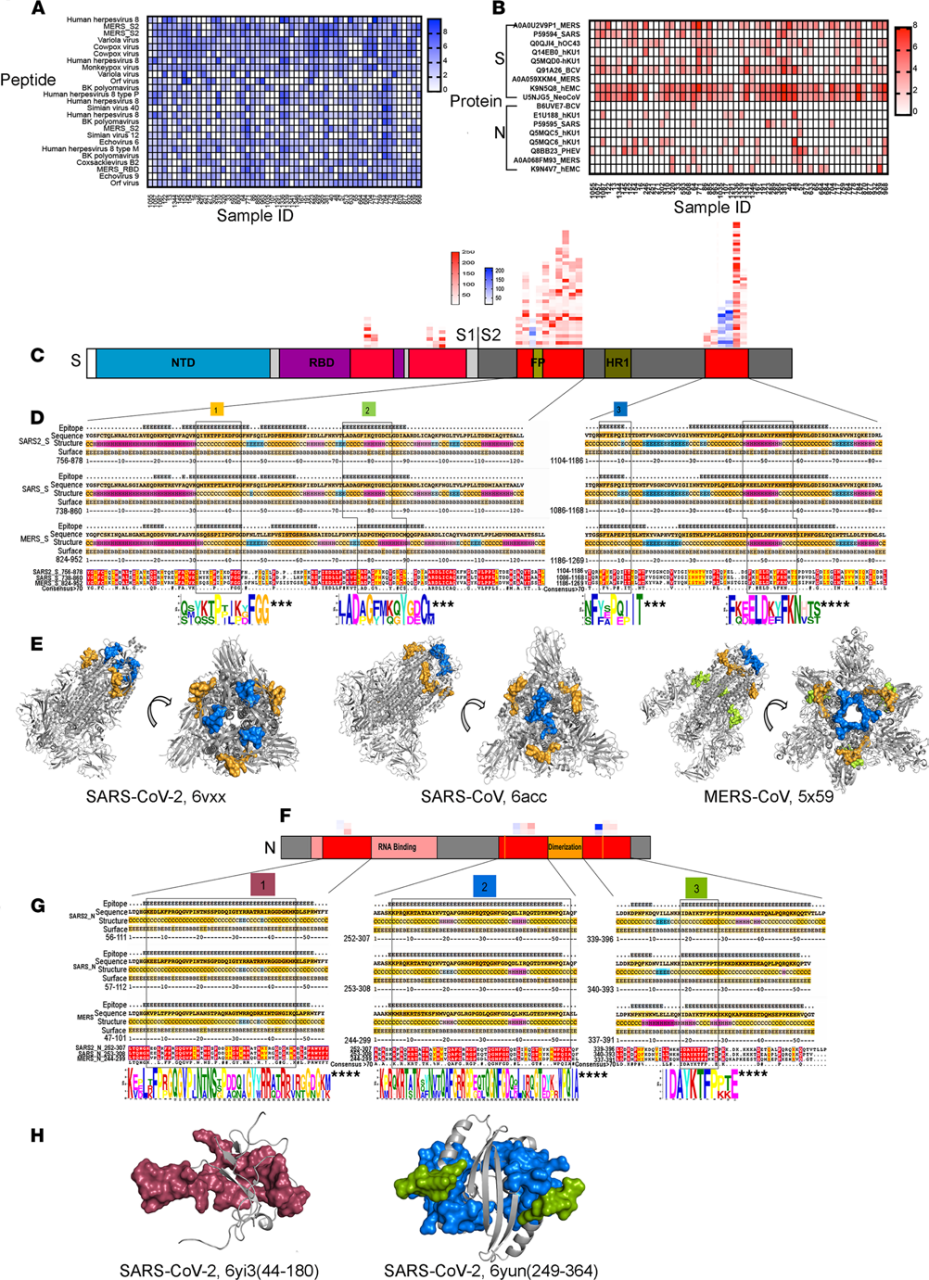
Linear epitope mapping of virus antibodies by VirScan. (**A**) The distribution of top 20 VirScan hit enrichments among 56 camel sera. (**B**) The hit enrichments of S and N proteins of all *Betacoronavirus* among 56 camel sera. (**C–E**) Sequence alignment and protein conformation analysis of S protein. (**C**) Schematic structure and VirScan hits of S protein. Red represents hits of MERS-CoV, and blue represents hits of SARS-CoV. (**D**) Overview of multiple alignment of antigenic regions of SARS-CoV-2, SARS-CoV, and MERS-CoV. Structure: helix (H; pink probability gradient), sheet (E; blue probability gradient), and coil (C, orange probability gradient) predicted using NetsurfP. Surface: buried(B)/exposed(E) from NetsurfP’s default threshold. Orange gradient illustrates predicted relative surface accessibility. ***E value < 0.001, ****E value < 0.0001, calculated by MEME. (**E**) S trimer of SARS-CoV-2, SARS-CoV, and MERS-CoV in the prefusion conformation. The view of conformation is observed from 2 directions, and the homology sequence position on protein is labeled by the corresponding colors. (**F–H**) Sequence alignment and protein conformation analysis of N protein. (**F**) Schematic structure and VirScan hits of N protein. (**G**) Overview of multiple alignment of antigenic regions of SARS-CoV-2, SARS-CoV, and MERS-CoV. (**H**) Monomer of the N protein of SARS-CoV-2 in the prefusion conformation. The structures of SARS-CoV and SARS-CoV-2 spanning region 2 in **D** and the structures of SARS-CoV and MERS-CoV spanning regions 1, 2, and 3 in **G** are not available in Protein Data Bank (PDB). Highlighted regions are shown as surfaces, whereas the protein backbone is shown as a cartoon. S proteins are represented using PDB codes 6ACC (SARS-CoV-2), 5X59 (MERS-CoV), and 6VXX (SARS-CoV-2), and N protein with PDB codes 6Y13 and 6YUN (SARS-CoV-2).

The microsphere-based SARS-CoV-2 competition/inhibition assay and the cell-based SARS-CoV-2 spike pseudovirus neutralization assay indicated the presence of SARS-CoV-2 spike-neutralizing antibodies induced by both conformational and linear epitopes. Although it is known that neutralizing antibodies react more often with conformational epitopes, several studies have revealed numerous linear epitopes of SARS-CoV-2 targeted by human neutralizing antibodies (26, 28, 29, 31). To reveal the large spectrum of linear epitopes targeted by the camel cross-reactive antibody repertoire, we used VirScan — a proteome-wide programmable phage display and phage immunoprecipitation-sequencing (PhIP-Seq) method that comprehensively identifies epitope-specific antiviral antibody repertoires against MERS-CoV and SARS-CoV (32, 33). SARS-CoV-2 peptides are not included in the current version of VirScan. We obtained a broadly diverse antibody repertoire targeting a myriad of camel pathogen viral peptides in 56 of the serum samples. Several peptides corresponding to MERS-CoV antigens and to many other animal viruses were enriched (Figure 4A). As expected, the enriched epitopes were indeed located in the S and N proteins; these proteins are involved in viral-host cell fusion and RNA replication, respectively, and are primary immunogenic targets for viral neutralization in *Betacoronaviruses*, including MERS, SARS-CoV, and SARS-CoV-2 (Figure 4B). Unexpectedly, most of the epitopes were concentrated in the S2 subunit of the S protein, while few were located in the S1 subunit (Figure 4C). Owing to the high degree of glycosylation, S1 protein peptides present on phage display might not capture all potential epitopes; however, 5 of the peptides from the S1 subunit were detected by VirScan (Figure 4C). To identify the conserved sequence motif, we performed multiple sequence alignment of highly enriched epitopes that shared linear sequence homology with the full-length proteins of SARS-CoV and SARS-CoV-2. Accordingly, we identified 4 enriched epitopes shared by the 3 coronaviruses. These epitopes were located in the S2 subunit, which is functionally essential and a highly conserved region of the spike protein (Figure 4D). The S2 subunit consists of a fusion peptide (FP), heptad repeat 1 and 2 (HR1 and HR2), a transmembrane domain, and a cytoplasmic fusion domain. These domains interact with each other to form a 6-helix bundle fusion core and are responsible for viral entry. The potential antibody-binding sites of both SARS-CoV and MERS-CoV were enriched in 2 regions, encompassing the FP and overlapping HR1, which share high identities among these 3 viruses (Figure 4D). These regions are positioned on the membrane fusion end of the S trimer structure (Figure 4E).

We also identified potential antibody-binding sites in the N protein, which comprises 2 distinct RNA-binding domains: the N-terminal domain (NTD) and the C-terminal dimerization domain (CTD) (Figure 4, F and G). These domains are interconnected by a weakly structured linkage region containing a serine-rich domain (Figure 4H).

### B cell epitope mapping of SARS-CoV-2 cross-reactive dromedary camel antibodies using a SARS-CoV-2 peptide/proteome microarray.

We used a recently validated SARS-CoV-2 peptide/proteome microarray to explore the repertoire of SARS-CoV-2–specific cross-reactive camel VHH antibodies (31, 34). We used different anti-camel Ig-isotype secondary antibodies specific for camel IgGs (all IgG isotypes) and IgG2/3s (VHH, single-chain antibodies) to compare the repertoire of linear B cell epitopes of the SARS-CoV-2 S1/S2 protein recognized by the different isotypes of camel MERS-CoV antibodies.

**Figure 5:**
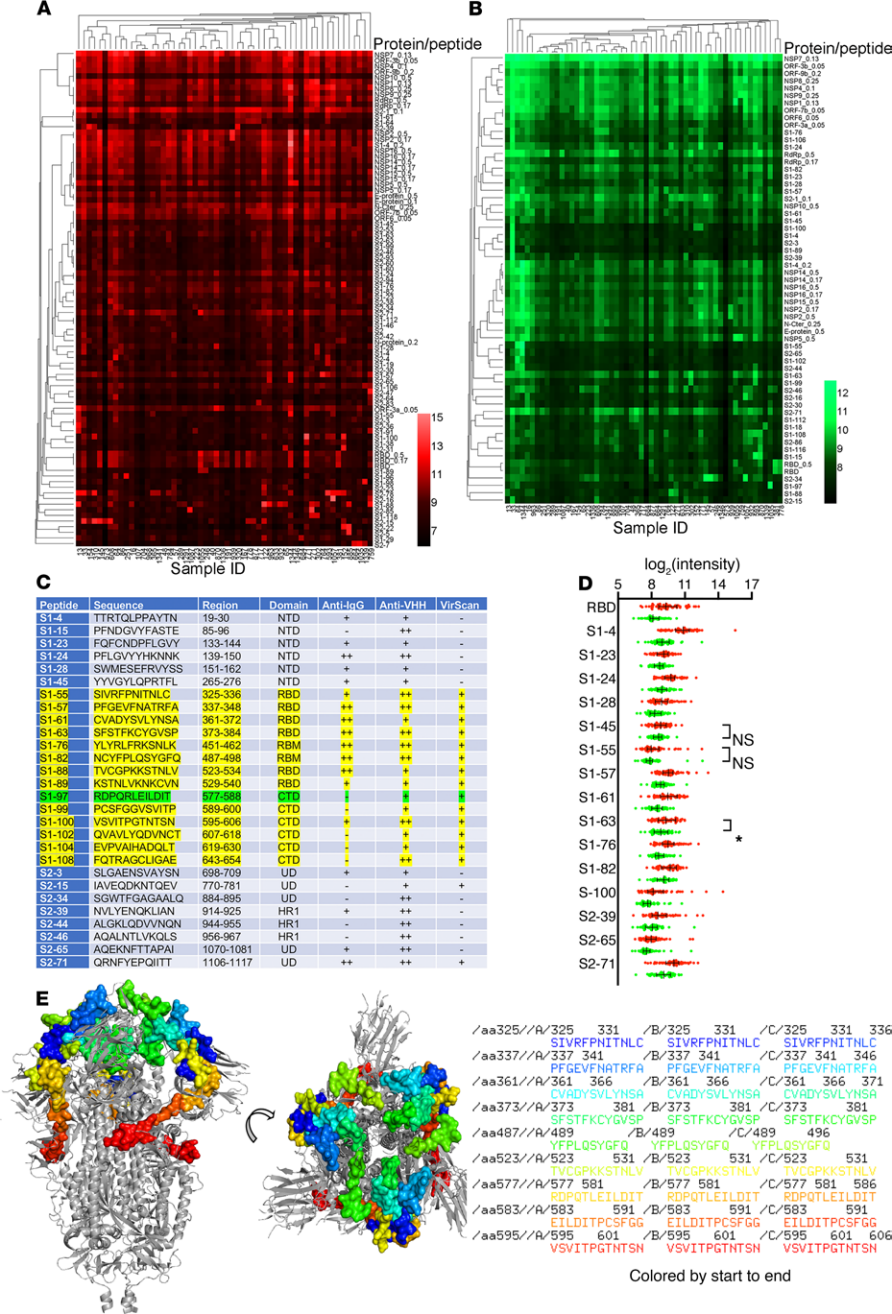
Identification of SARS-CoV-2 peptide– and protein-specific antibodies by SARS-CoV-2 peptide/proteome array. Enriched hits SARS-CoV-2–specific (**A**) total IgG and (**B**) heavy-chain only IgG and VHH profiles of 56 camel sera. Each square indicates the camel serum (columns) reactivity to the peptides and proteins (rows) of SARS-CoV-2. Mean fluorescence intensity (MFI) is shown by a color gradient scale. (**C**) SARS-CoV-2 S1/S2 linear epitopes recognized by camel VHH antibodies. In the VirScan column, “+” indicates that the sequence is homologous with SARS-CoV and/or MERS-CoV VirScan hits. NTD, N-terminal domain; RBD, receptor-binding domain; RBM, receptor-binding motif; CTD, C-terminal domain; UD, undefined; HR1, heptad repeat 1. Epitopes found highly immunogenic in humans are highlighted in yellow. The S1-97 peptide, found as a neutralizing epitope in humans, is highlighted in green. (**D**) The comparison of levels (mean with interquartile range) of shared hits by total IgG (red) and VHH (green) antibodies among 56 camel sera using Student’s *t* test. *P < 0.05. (**E**) Selected S1 hits, which were revealed by both anti-total IgG and anti-VHH antibodies, on a structure of S trimer of SARS-CoV-2.

Fifty-six serum samples from MERS-CoV–seropositive dromedaries and the appropriate controls were probed in the SARS-CoV-2 peptide/proteome microarray. After data filtering and normalization, we built a camel IgG and VHH profile for each serum sample and performed cluster analysis to generate heatmaps of the enriched hits for visualization (Figure 5, A and B). The MERS-CoV–seropositive serum samples and controls were perfectly clustered for both the camel IgG and VHH antibodies, attesting to the specificity of the SARS-CoV-2 peptide/proteome microarray (Figure 5, A and B). In agreement with the VirScan analysis, we found variable specificities among the 56 tested camel serum samples, but there was remarkable cross-reactivity between the camel IgG and VHH antibodies and the full-length SARS-CoV-2 proteins (Table 2). Several SARS-CoV-2 nonstructural proteins (e.g., NSP1, -2, -7, and -8, and ORF6 and ORF7) elicited marked cross-reactivity with both camel Ig isotypes (Table 2). NSP14, ORF6, ORF7b, and the S2 subunit of the S protein were predominantly targeted by camel IgG1 rather than by VHH antibodies (Table 2). Strikingly, there was strong IgG1 and VHH antibody reactivity against NSP7, NSP8, and the RNA-dependent RNA polymerase (RdRp) of SARS-CoV-2 (nsp12) (Table 2). The RdRp of SARS-CoV-2, consisting of the nsp12 catalytic subunit and the nsp7 and nsp8 cofactors, is a key component of the replication/transcription machinery (35). In addition, variable specificities and considerable cross-reactivity against the S1 NTD, RBD, and CTD and the S2 HR1 and HR2 domains was revealed (Table 2). A list of S1/S2 peptides targeted by VHH isotypes is presented in Figure 5C. Interestingly, 12 SARS-CoV-2 S1 peptides that reacted with camel antibodies were found to be highly immunogenic in humans. Notably, 8 of the 11 camel serum samples showing high SARS-CoV-2 cross-neutralizing antibody activity reacted with one or more linear RBD peptides (Table 3); S1-82 reacted with 5 SARS-CoV-2–neutralizing camel serum samples; S1-61 and S1-64 reacted with 4 samples; and S1-57, S1-63, and S1-76 reacted with 3 samples. Although it is known that neutralizing antibodies react more often with conformational epitopes, these RBD peptides, targeted by multiple cross-neutralizing serum samples, could be SARS-CoV-2–neutralizing epitopes. Notably, the linear epitopes that we identified (e.g., S1-76/97) are not only highly immunogenic in humans but are also physiologically relevant because they have been identified in patients with COVID-19 (31). Therefore, these epitopes could serve as promising candidates for the development of broadly neutralizing antibodies. Epitopes revealed exclusively by the camel antibodies could increase the pool of neutralizing antibodies with potential therapeutic use.

By comparing the total IgG signal and VHH signal from the same antigens, we found that antibodies recognizing the S1-45, S1-55, and S1-63 epitopes might all belong to the VHH subclass (Figure 5D). We also located the identified epitopes on the S trimer structure (Figure 5E).

## Discussion

Our study showed the presence of SARS-CoV-2 cross-reactive and cross-neutralizing antibodies in SARS-CoV-2–nonimmunized dromedary camels and provides a comprehensive structural analysis of the targeted SARS-CoV-2 proteins and linear epitopes. Because the titers of these SARS-CoV-2 cross-neutralizing camel antibodies were not found consistently and to be exceptionally high, they cannot be proposed as a potential treatment for COVID-19. Rather, their presence in nonimmunized camels suggests that these dromedaries might produce highly efficient antibodies once they are actively immunized with SARS-CoV-2 antigens.

The camel cross-reactive antibody epitope mapping revealed not only several epitopes known to be highly immunogenic in humans, including a neutralizing epitope (31), but also epitopes exclusively targeted by camel antibodies. The identified highly immunogenic SARS-CoV-2 epitopes could be used as immunogens to develop SARS-CoV-2 hyperimmune camels.

One short-term implication of these findings is that after actively immunizing camels with SARS-CoV-2 antigens, the SARS-CoV-2 hyperimmune dromedaries could generate novel COVID-19 serotherapy tools to complement or replace the current CCP therapy. Given the total blood volume of a camel and the large herd size of dromedary camels living in the Middle East and North African regions, camel plasma would be available in quantities sufficient to meet the needs of a large population. The proposed SARS-CoV-2 hyperimmune camel plasma–based COVID-19 serotherapy could be used as a longer-term treatment option, particularly in low- and middle-income countries where resource constraints could bar access to novel treatments (e.g., COVID-19 vaccines), even if they become widely available. Similar to any immunoglobulin-based treatment, the proposed camel serum–based therapy for COVID-19 treatment should overcome potential pitfalls such as the exaggerated inflammatory response seen in the antibody-dependent enhancement process (36). Additionally, after binding to the viral immune complexes, subneutralizing antibodies could bind to FcγR-bearing cells, leading to increased viral uptake and replication (37).

Numerous human SARS-CoV-2–neutralizing antibodies have been recently reported; however, their binding affinities and pseudoviral- and viral-neutralizing abilities have varied (25–30, 38–40). Liu et al. highlighted the short-duration protective effect of human SARS-CoV-2–neutralizing antibodies and raised concerns about the efficacy of future SARS-CoV-2 vaccines (41). In contrast to human antibodies, camel anti–MERS-CoV–neutralizing antibodies, which were shown to cross-react with SARS-CoV-2, persist several years after infection. This could be because of repeated exposures to the virus, but some studies indicate that these camel antibodies have long-lasting efficacy (17–19). It remains unclear whether this durable effect arises from the structural and/or functional features of camel antibodies or from some other component of the camel plasma. Relatedly, adverse reactions in humans to animal-derived polyclonal antibodies are usually due to the presence of highly immunogenic animal proteins. This is less likely to occur with camel serum–based therapy, because camel IgGs are less immunogenic than most mammalian IgGs, and when administered intravenously, they are less likely to induce serum sickness and anaphylactic adverse reactions (42, 43). Growing research in the field of antibody engineering has focused on enhancing the therapeutic efficacy of VHHs. Strategies that enable VHHs to cross the blood-brain barrier have recently shown promise. These findings have driven tremendous growth in the use of VHHs for treating central nervous system diseases (44–46). A limiting factor for the clinical use of VHH domains is that the hydrodynamic radius of VHHs falls below the kidney’s glomerular filtration limit, which can contribute to rapid renal clearance and a weak pharmacokinetic (PK) profile, considerably affecting their therapeutic effectiveness. To address this issue, several antibody engineering techniques have been employed to increase VHH size and improve the PK profile. The simple formation of genetic fusions has frequently been used, for example, with conventional Fc (47), VHH repeat domains (mono- or polyspecific), serum proteins (e.g., HSA), and anti-serum albumin VHH (48). As an alternative to genetic fusions and modularity, chemical methods, such as PEGylation and lipidation, have been applied to increase the VHH half-life (49–51).

Dromedary camels may constitute a competitive source for the development of COVID-19–targeted antibody therapy. Interest has been growing in recent years in the generation and use of camel single-chain antibodies (VHHs or nanobodies) and their derivatives for a wide spectrum of applications (52, 53). The therapeutic properties of camel VHHs can be enhanced by protein engineering to improve their efficacy (54, 55). The use of camel nanobodies is of special interest for the recognition of epitopes that are usually not antigenic for conventional antibodies. In the case of COVID-19, the unique feature of VHHs to easily penetrate tissues, including the lungs (the main target of SARS-CoV-2), gives them additional potential curative properties to treat SARS-CoV-2 infection. Camel nanoantibodies might be an appropriate choice for generating a COVID-19 treatment, because these single-chain antibodies are highly soluble, small, and stable proteins and can be produced in large quantities (52).

Recent studies have found that SARS-CoV-2–nonexposed individuals have cross-reactive antibodies to a number of coronaviruses, including SARS-CoV-2 (56, 57). Particularly, Ng et al. found that in SARS-CoV-2–nonexposed individuals possess neutralizing antibodies targeted to the S2 protein (58). Moreover, many of these cross-reactive antibodies found in humans are unique to FP epitopes or adjacent S2 subunit epitopes, which are suggested to neutralize the coronaviruses by blocking viral membrane fusion and host cell entry (59). Importantly, recent studies have shown that antibodies induced by the active immunization of llamas with MERS-CoV and SARS-CoV viral antigens (in particular, SARS VHH-72) cross-react with the spike protein of SARS-CoV-2 (60, 61). Although dromedary camels and llamas belong to the same Camelidae family and their antibodies share the single-chain antibody feature, the lack of natural infection in llamas by MERS-CoV means they cannot replicate the efficient response of dromedary camels to MERS-CoV infection. Dromedaries experience rapid viral clearance without showing any disease symptoms. This could arise from innate immunity, efficient neutralizing antibodies, or other antiviral immunity components, providing support for the use of dromedaries in the development of COVID-19–targeted antibody therapies. Indeed, in a recent study, sera from dromedary camels that was seropositive for MERS-CoV was highly efficient when administered to mice infected with MERS-CoV (62). Camel serum given both before and after exposure protected the infected mice from weight loss, diminished the histological changes in the lungs, and accelerated viral clearance (62). Moreover, the low 12–30 kDa MW of nanobodies also offers new and noninvasive routes of administration, such as delivery by inhalation, which has been proven in clinical trials to be safe and successful in preventing respiratory syncytial virus infection (63, 64). Numerous nanobodies are being investigated in clinical trials (65), and one nanobody (caplacizumab) has already been approved by the US Food and Drug Administration for the treatment of thrombotic thrombocytopenic purpura (66). Additionally, camel milk, with its unique nutritional composition and abundance of secreted IgA and VHH nanobodies (67), has potential implications for the induction of passive immunity to SARS-CoV-2. Immunizing lactating camels with SARS-CoV-2 antigens might induce SARS-CoV-2–neutralizing antibodies in camel serum and milk.

In summary, we identified the presence of SARS-CoV-2 cross-reactive neutralizing antibodies in dromedary camels that were not previously immunized with SARS-CoV-2 antigens and have revealed the structures of the corresponding major target linear B cell epitopes. Our findings advocate for the development of SARS-CoV-2 hyperimmune camels as a prominent source of therapeutic agents for the prevention and treatment of COVID-19. With adequate testing and clinical trials, the proposed SARS-CoV-2 camel serum–based serotherapy could have a major impact as a preventive and curative intervention for COVID-19. By taking advantage of the unique features of the camel immune system, the suggested intervention might provide protective passive immunization in humans before and after exposure to SARS-CoV-2 and in patients with established disease, thus helping alleviate the burden of the current pandemic.

## Methods

### Dromedary camel sample collection.

Sera from 229 dromedary camels was collected from 2 camel cohorts in Qatar. All camels were female, with an age range of 4–15 years. A total of 129 serum samples were collected from a camel slaughterhouse before September 2019; the other 100 samples were taken from live camels by jugular puncture for routine infection screening in 2020. All samples were stored at −80°C until testing. The Ministry of Public Health has extensively tested dromedaries in Qatar, including the living camels enrolled in the present study, for SARS-CoV-2 infection using nasal swab sampling, and no positive cases have been reported.

### Seroconversion assay.

The camel serum samples were tested for the presence of antibodies binding SARS-CoV-2 M, N, S trimer, E, and MERS-CoV S proteins using a laboratory-made bead array. The following Spherotech carboxyl microspheres were used: 10^7^ microspheres of peaks 8, 6, 4, and 2 from the blue particle array kit (Spherotech, CPACK-5067) and peak 11 from the UV particle array kit (Spherotech, UVCPACK-5042-1). The microspheres were washed once in diH_2_O and activated in the presence of 80 mM monobasic sodium phosphate, pH 6.2, 5 mg/ml sulfo-NHS (Pierce, 24520), and 5 mg/ml EDC (Pierce, 77149) under shaking for 20 minutes at room temperature. The activated microspheres were then washed 3 times with PBS, pH 7.4. Blue peaks 8, 6, 4, and 2 and UV peak 11 were respectively incubated with 100 μg of recombinant SARS-CoV-2 E, S trimer, N, M, and MERS-CoV S1 under rotation overnight at room temperature (Supplemental Table 1; supplemental material available online with this article; https://doi.org/10.1172/jci.insight.145785DS1). Finally, the microspheres were washed twice with PBS-TBN (0.2% Tween-20, 0.1% BSA, 0.05% sodium azide) and stored at 4°C in PBS-TBN until further use. To assess the antibody titers, the serum samples were diluted 20-fold (initial dilution followed by serial dilution) in assay buffer (10 mM Tris-HCl, pH 7.5, 0.1% BSA, 0.01% Tween-20) and incubated with the SARS-CoV-2 M, N, S trimer, E, and MERS-CoV S1 microspheres (2500 microspheres for each peak) under shaking for 1 hour at room temperature in a Multiscreen HV filter plate (Millipore, MSHVN4510). After 3 vacuum washes in assay buffer, the microspheres were incubated in 1 μg/ml goat anti-IgG camel antibody (ADI, 30835-UL) conjugated in-house to Alexa Fluor 594 (AF594) or in 1 μg/ml anti-camelid VHH antibodies conjugated to Phycoerythrin (PE) (GenScript, A02018). These incubations were conducted in assay buffer, with shaking for 30 minutes at room temperature. The microspheres were then vacuum washed 3 times in wash buffer (10 mM Tris-HCl, pH 7.5, 0.05% Tween-20), resuspended in the same buffer, and analyzed in a BD FACS Symphony A5. The flow cytometry experiment analyzed each bead region with the UV and blue laser, and the detection antibody was analyzed with the yellow-green laser. To assess the participation of VHH antibodies in the overall camel seroconversion, 1 μg/ml iFluor647-labeled anti-VHH cocktail antibody (GenScript, A02019) was used to detect overall seroconversion. The data were analyzed using FlowJo software; each bead region was gated to measure antibody binding. A minimum of 100 beads per region was acquired. The median fluorescence intensity (MFI) of each bead set was used in the subsequent calculations.

### In vitro competition/inhibition assay.

The neutralization activity of the camel sera against SARS-CoV-2 was tested using an in vitro competition/inhibition assay. A standard inhibition curve was first prepared from a standard solution of a SARS-CoV-2 RBD human neutralizing antibody isolated from a SARS-CoV-2–infected patient (Acro Biosystems, SAD-S35). The curve started at 5 μg/ml and proceeded in a 12-step, 2-fold dilution series in assay buffer. The camel sera were serially diluted (1:2, 1:6, 1:18, 1:54, 1:162, and 1:486) in assay buffer. Next, 0.5 μg/ml biotinylated human ACE2 (Acro Biosystems, AC2-H82F9) was added to the neutralizing antibody standards and to the camel sera dilutions. Mixed samples with ACE2 were then added to the SARS-CoV-2-S1 and SARS-CoV-2-RBD microspheres (2500 microspheres for each peak) (Supplemental Table 1) and incubated with shaking for 45 minutes at room temperature. The microspheres were then washed 3 times with assay buffer and incubated in 4 μg/ml streptavidin-PE with shaking for 20 minutes at room temperature. After 3 washes in wash buffer, the microspheres were resuspended in the same buffer and detected in a BD FACS Symphony A5. The competition for binding to RBD between the RBD-specific human IgG1 monoclonal antibody AS35 (Acro Biosystems, SAD-S35) and camel serum samples was assayed using RBD-coupled microspheres. 2500 RBD-coupled microspheres in 50 μl were incubated for 45 minutes with a 50 μl mixture of AS35 at 0.15 μg/ml and 2-fold serially diluted camel serum samples from 1:10 to 1:1280 in a MultiScreen filter plate (Millipore, MSHVN4510). After 3 vacuum washes, camel antibody binding on RBD was revealed by goat anti-camel IgG antibody (ADI, 30835-UL) conjugated to AF594 and the binding of the human neutralizing antibody was revealed with AF488-labeled goat anti-human anti-IgG1 antibodies (SouthernBiotech, 9052-30). The maximum binding (MFImax) was determined as the MFI read on beads incubated only with the human neutralizing antibody. Inhibition of binding at a given dilution of camel serum was calculated by using the median fluorescent intensity (MFIdil) in the following formula: percentage inhibition = (1 – [MFIdil/MFImax] × 100. The inhibition of neutralizing antibody binding on RBD and camel serum binding on RBD were calculated at different serum dilution factors. To investigate the participation of VHH antibodies in neutralizing S trimer binding to ACE2, the competition/inhibition assay was performed as described above until the detection step, which was accomplished with a mix of iFluor647-labeled anti-VHH cocktail antibody (GenScript, A02019) and streptavidin-PE to concomitantly reveal the binding of VHH antibodies and hACE2 to RBD or SARS-CoV-2 S1 proteins. The data were analyzed using FlowJo software. A minimum of 100 beads per region was acquired. The MFI of each bead set was used in the subsequent calculations.

### Pseudovirus neutralization assay.

A SARS-CoV-2 pseudovirus neutralization assay kit (GenScript, SC2087A) was used to evaluate the ability of the camel sera to block cell entry of the pseudotyped lentiviral particle of SARS-CoV-2 spike. HEK293 cells overexpressing ACE2 (HEK293-ACE2 cells) were seeded into 96-well plates and infected with 50 μL of the pseudotyped luciferase SARS-CoV-2 spike with or without diluted camel serum. ACE2-Fc was used as a positive control, and serum from a healthy person was used as a negative control. After 6 hours of incubation at 37°C, the pseudovirus-containing medium was replaced with fresh cell culture medium and the plate was incubated for another 48 hours. After removing the culture media, Bio-Glo luciferase substrate working solution (Promega, G7940) was added to the HEK293-ACE2 cells. Luciferase activity, expressed in relative luminescence units, was measured with an EnVision plate reader.

### VirScan analysis.

Serological profiling of the camel serum antiviral IgG repertoire was performed using PhIP-Seq as described by Xu et al. (32). Briefly, the VirScan 2.0 library was programmed into an Agilent microarray and was then amplified and ligated into bacteriophage T7 DNA, packaged into phage particles, and amplified in *E*. *coli*. The amplified libraries were incubated with 2 μL camel serum at 4°C overnight with protein A and G magnetic beads (Invitrogen). Antibody-bound phage were immunoprecipitated, and balanced amplicon libraries were pooled and sequenced. The read counts per peptide were converted to a relative antibody epitope binding signal, and the magnitude was reported as an epitope-specific *z* score.

### Protein/peptide microarray.

Microarray-based serum analysis of anti–SARS-CoV-2 antibodies in the camel serum was performed as previously described (31, 34). The arrays were detected by incubating with APC-conjugated goat anti-camel IgG antibodies (Alpha Diagnostic, 30385) and iFluor555-conjugated anti-camelid VHH antibodies (GenScript, A01863). The fluorescence signals were scanned by a LuxScan 10K-A (CapitalBio Corporation), and the fluorescence intensity data were extracted by GenePix Pro 6.0 software (Molecular Devices).

### Bioinformatics analysis.

Multiple sequences were aligned by Clustal Omega on an EBI server or by T-Coffee (68) and illustrated using ESPript 3.0 (69). Linear B cell epitopes with a threshold greater than 0.5 were predicted by BepiPred-2.0 and are displayed with an orange gradient (Figure 4, D and G) (70). The common motif was calculated and illustrated by MEME (71). The secondary structures of helix, sheet, and coil were predicted using NetsurfP (72). Structure visualization was conducted using PDB files downloaded from the Protein Data Bank (PDB) in Europe (https://www.ebi.ac.uk/pdbe) and PyMOL 2.4 (https://pymol.org/2/).

### Statistics.

All statistical analyses were performed using GraphPad Prism 7. Statistical parameters are reported in the figure legends. An unpaired or paired 2-tailed Student’s *t* test was used for 2-group comparisons for normally distributed data. An asymmetric sigmoidal 5-parameter logistic model was used to generate standard curve for in vitro competition/inhibition assay. A typical 4-parameter logistic model was used to fit dose (dilution) versus response (inhibition percentage) curves to the data of camel serum samples. The correlation between inhibition percentage and fluorescence signal was analyzed by linear regression. *P* values of less than 0.05 were considered significant.

### Study approval.

The study was approved by the Institutional Animal Care and Use Committee of Weill Cornell Medicine–Qatar.

## Author contributions

LC designed the study. LC, JS, JCG, MS, SWJA, EABAF, and MHAT performed experiments and collected the data. JCG, IP, SM, and AS performed in vitro binding and neutralization assay. NM, MMAA, HEAR, and AIC generated and interpreted the data. LC, JS, MS, JCG, SR, and AR analyzed and interpreted the data. NH generated the epitope 3-dimensional structure confirmation data. LC, MS, and JS wrote the manuscript, did the literature search, and analyzed and interpreted the data. LC supervised and coordinated the study. All authors contributed to reviewing and approved the final version

## Supplementary Material

Supplemental Table 1

## Figures and Tables

**Table 1:**
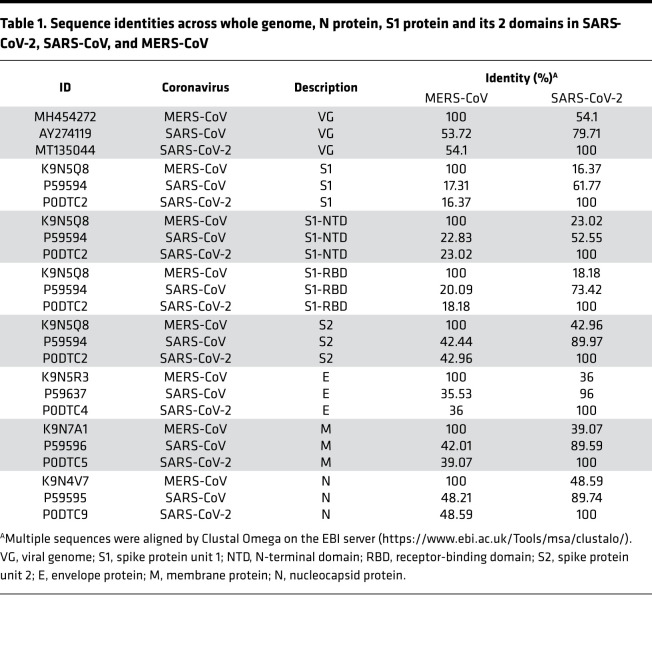
Sequence identities across whole genome, N protein, S1 protein and its 2 domains in SARS-CoV-2, SARS-CoV, and MERS-CoV

**Table 2:**
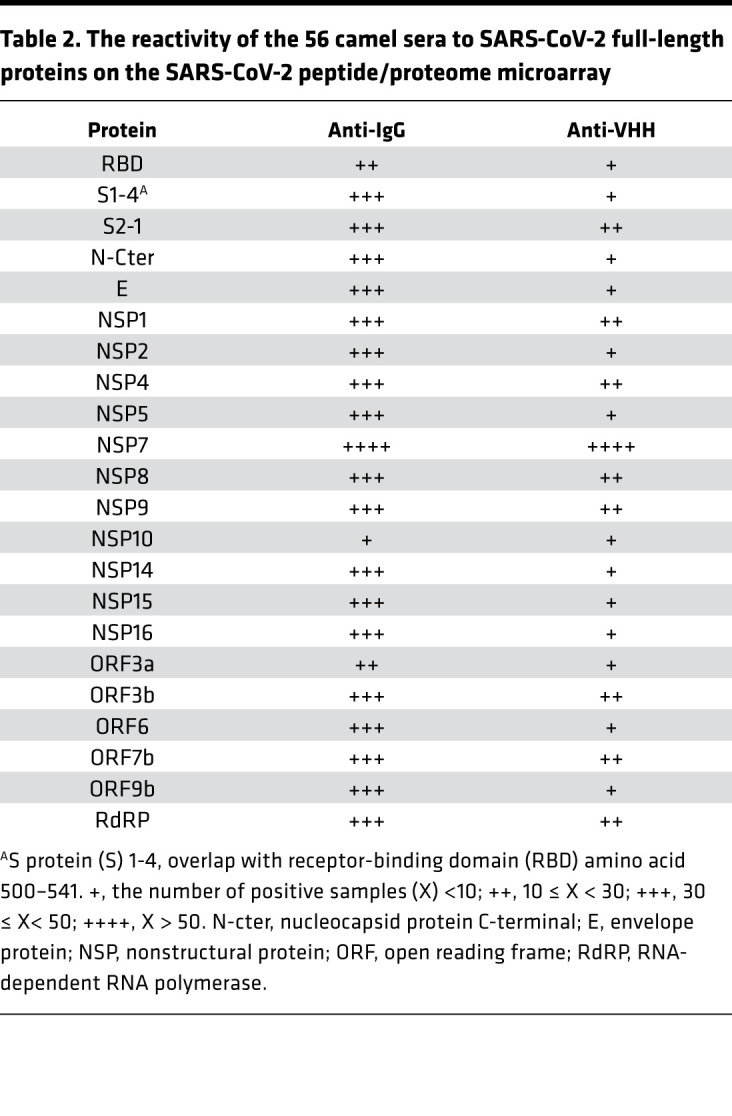
The reactivity of the 56 camel sera to SARS-CoV-2 full-length proteins on the SARS-CoV-2 peptide/proteome microarray

**Table 3:**
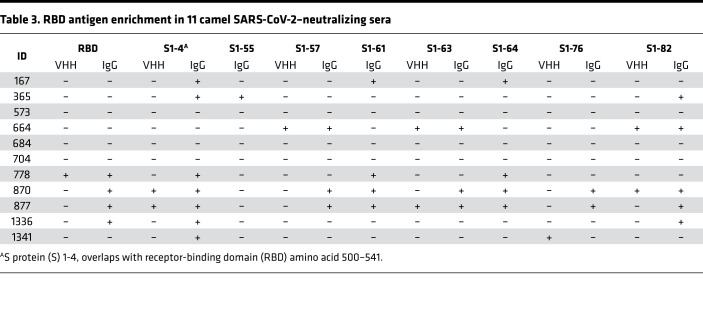
RBD antigen enrichment in 11 camel SARS-CoV-2–neutralizing sera

## Version 1 02/02/2021

In-Press Preview

## Version 2 03/08/2021

Electronic publication
