# Supplementary material for: Dromedary camels as a natural source of neutralizing nanobodies against SARS-CoV-2
Source: JCI Insight. 2021 Mar 8;6(5):e145785. doi: 10.1172/jci.insight.145785 (PMC8021111; doi:10.1172/jci.insight.145785)
Supplement: Supplemental Table 1 [file jciinsight-6-145785-s180.pdf]

Dromedary camels as a natural source of neutralizing nanobodies against SARS-CoV-2

Lotfi Chouchane[1,2,3], Jean-Charles Grivel[4], Elmoubasher Abu Baker Abd Farag[5], Igor Pavlovski[4], Selma Maacha[4], Abbirami Sathappan[4], Hamad Eid Al-Romaihi[5], Sirin W J Abuaqel [1,2,3], Manar Mahmoud Ahmad Ata[6], Aouatef Ismail Chouchane[6], Sami Remadi[7], Najeeb Halabi [2,3], Arash Rafii [2,3], Mohammed H Al-Thani[8], Nico Marr[6], Murugan Subramanian[1,2], Jingxuan Shan[2,3]*

[1] Department of Microbiology and Immunology, Weill Cornell Medicine, New York, USA.

[2] Genetic Intelligence Laboratory, Weill Cornell Medicine-Qatar, Qatar Foundation, Doha, Qatar.

[3] Department of Genetic Medicine, Weill Cornell Medicine, New York, USA.

[4] Deep Phenotyping Core, Research Branch, Sidra Medicine, Doha, Qatar.

[5] Department of Communicable Diseases Control, Ministry of Public Health, Doha, Qatar.

[6] Department of Immunology, Research Branch, Sidra Medicine, Doha, Qatar

[7] Laboratoire CYTOPATH, Sousse, Tunisia

[8] Ministry of Public Health, Doha, Qatar.

[*] **Corresponding Author**: Dr. Lotfi Chouchane, Weill Cornell Medicine, 445 East 69th Street Suite 432, New York, NY 10021; Phone: 646-962-4953. FAX: 646-962-4960, Email: loc2008@med.cornell.edu

Table S1. Proteins used for flow cytometry and immunofluorescence

Microsphere	Protein Name	Catalog#
Blue Peak 2	SARS-CoV-2-M	MBS8574735
Blue Peak 4	SARS-CoV-2-Nucleocapsid	NUN-C5227
Blue Peak 6	SARS-CoV-2-S-Trimer	SPN-C52H8
Blue Peak 8	SARS-CoV-2-Envelope	MBS8574736
UV Peak 9	SARS-CoV2-S1	S1N-C52H4
UV Peak 10	SARS-CoV2-RBD	SPD-C52H3
UV Peak 11	MERS-CoV-S1	40069-V08H
